# Hybrid two-stage CNN for detection and staging of periodontitis on panoramic radiographs

**DOI:** 10.1016/j.jobcr.2025.08.019

**Published:** 2025-08-28

**Authors:** Rini Widyaningrum, Eha Renwi Astuti, Adioro Soetojo, Amalia Nur Faadiya, Aga Satria Nurrachman, Netya Dzihni Kinanggit, Abdul Harits Iftikar Nasution

**Affiliations:** a Oral and Maxillofacial Radiology Specialist Study Program, Faculty of Dental Medicine, Universitas Airlangga, Jl. Mayjen Prof. Dr. Moestopo No.47, Surabaya, 60132, Indonesia; b Department of Dentomaxillofacial Radiology, Faculty of Dentistry, Universitas Gadjah Mada, Jl. Denta No.1, Sekip Utara, Yogyakarta, 55281, Indonesia; c Department of Conservative Dentistry, Faculty of Dental Medicine, Universitas Airlangga, Jl. Mayjen Prof. Dr. Moestopo No.47, Surabaya, 60132, Indonesia; d Dental Study Program, Faculty of Dentistry, Universitas Gadjah Mada, Jl. Denta No.1, Sekip Utara, Yogyakarta, 55281, Indonesia; e Master of Biomedical Engineering, Universitas Gadjah Mada, Jl. Teknika Utara, Pogung, Sinduadi, Mlati, Sleman, Yogyakarta, 55284, Indonesia; f Neura Integrasi Solusi, Jl. Kebun Raya No. 73, Rejowinangun, Kotagede, Yogyakarta, 55171, Indonesia

**Keywords:** Periodontal disease, Staging, Panoramic, Deep learning, Early detection, Radiograph, Medicine

## Abstract

**Background:**

Periodontal disease is an inflammatory condition causing chronic damage to the tooth-supporting connective tissues, leading to tooth loss in adults. Diagnosing periodontitis requires clinical and radiographic examinations, with panoramic radiographs crucial in identifying and assessing its severity and staging. Convolutional Neural Networks (CNNs), a deep learning method for visual data analysis, and Dense Convolutional Networks (DenseNet), which utilize direct feed-forward connections between layers, enable high-performance computer vision tasks with reduced computational demands. This study aims to evaluate the performance of a hybrid two-stage CNN integrating Mask R-CNN with DenseNet169 for detecting and staging periodontitis in panoramic radiographs.

**Methods:**

A total of 600 panoramic radiographs were divided into training (70 %), validation (10 %), and testing (20 %) datasets, with an additional 100 external radiographs used as a final testing set. Four types of annotations were applied: tooth segmentation, radiographic bone loss (RBL), cementoenamel junction (CEJ) area, and periodontitis staging (normal, stage 1, 2, 3, and 4). Mask R-CNN was employed for segmentation training to detect teeth, CEJ, and RBL, while DenseNet169 served as the classifier for periodontitis staging.

**Results:**

The hybrid two-stage CNN achieved a periodontitis staging performance on the external testing set with specificity and accuracy of 0.88 and 0.80, respectively.

**Conclusion:**

These results demonstrate the potential of this hybrid two-stage CNN model as a diagnostic aid for periodontitis in panoramic radiographs. Further development of this approach could enhance its clinical applicability and accuracy.

## Highlights

• Clinical and radiographic evaluation, including panoramic radiographs, is essential for diagnosing and staging periodontitis.
• A hybrid Two-Stage CNN with Mask R-CNN and DenseNet169 effectively detects and stages periodontitis on panoramic radiographs.
• The proposed deep learning model achieved an accuracy of 0.80 for periodontitis staging on an external dataset of 100 radiographs.

## Graphical abstract

**Figure:**
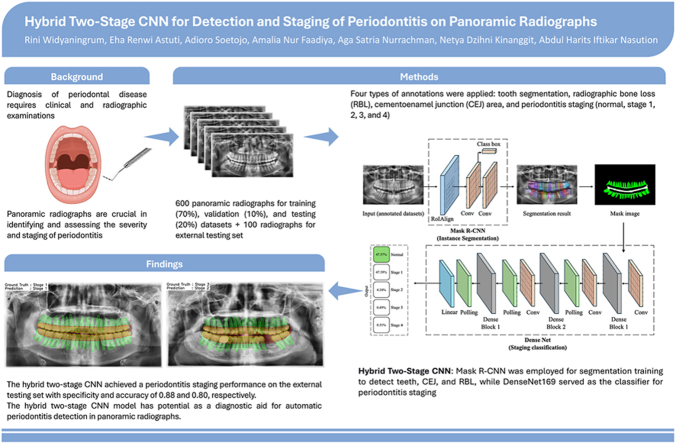

## 1 Introduction

Periodontitis is a chronic inflammatory disease that affects the connective tissues supporting the teeth, causing damage to the periodontal ligament and alveolar bone. The condition is associated with systemic health issues and can impact the upper and lower jaw, leading to tooth loss in adults.^1^, ^2^, ^3^ Affecting up to 10 % of the global population, early detection and diagnosis are crucial for effective management of periodontitis.^1^^,^^2^

Alveolar bone loss is a key indicator of periodontitis that can be observed in radiographs.^4^, ^5^, ^6^ Radiographs reveal bone loss, caries, furcation defects, subgingival calculus, and other pathological conditions in periodontal tissues. Radiographic bone measurements correlate significantly with clinical attachment loss (CAL) from probing.^4^^,^^5^ Thus, radiographs provide critical information about root and bone conditions, supporting clinical decisions and confirming periodontitis alongside CAL, periodontal pockets, and gingival bleeding.^7^

Radiographic examination is critical in diagnosing, assessing, and managing periodontal disease, aiding systemic health prevention, and improving quality of life.^1^^,^^8^ While periapical radiographs are commonly used to assess radiographic bone loss (RBL), panoramic radiographs can also effectively diagnose and evaluate periodontal disease.^9^ The RBL measurement on a radiograph can be done manually or using a computerized diagnostic support system.^6^ Periapical radiographs demonstrate higher accuracy in detecting bone defects and marginal bone levels due to their detailed imaging, making them ideal for assessing furcation involvement. Conversely, panoramic radiographs provide a broader view of dental arches but may overestimate bone loss and exhibit greater distortion.^10^ While periapical radiographs require multiple exposures for wider coverage, panoramic radiographs are more efficient for initial evaluations and long-term monitoring of extensive bone changes.^11^

Periodontitis classification follows the 2017 World Workshop on the Classification of Periodontal and Peri-Implant Diseases and Conditions, utilizing staging and grading systems based on CAL, RBL, tooth loss, case complexity, and disease extent.^7^ The staging of periodontitis indicates the severity of the supporting tissue condition around the teeth and is used to determine care management for individuals with periodontitis.^12^ Meanwhile, the grading system categorizes periodontitis as slow, moderate, or rapid progression based on bone loss relative to age.^7^

Artificial intelligence (AI) has improved radiodiagnosis in oral radiology by addressing the unreliability of manual measurements.^9^ AI can serve as an automated diagnostic support system, reducing human error, providing more accurate information, and preventing misdiagnosis caused by inexperience or fatigue. Additionally, AI enhances efficiency, lowers costs, and enables faster medical data analysis.^13^ In radiology, AI-driven image analysis is a critical study area in computer vision.^14^ AI technology is primarily built using machine learning (ML) and deep learning (DL), where ML employs self-training algorithms constructed from datasets to predict new information. At the same time, DL, a subset of ML, mimics human brain functionality through artificial neural networks for learning from large datasets.^14^^,^^15^ Deep convolutional neural networks (CNNs) are the most commonly used for detecting RBL and classifying periodontitis in panoramic radiographs, utilizing references like the 2017 World Workshop on Periodontal Classification^12^^,^^16^, ^17^, ^18^, ^19^ and the WHO's Community Periodontal Index (CPI).^15^^,^^20^^,^^21^

In dentistry, CNNs are used for image segmentation, detection, classification, and quality enhancement.^15^^,^^22^^,^^23^ CNNs, particularly regional models like Faster R-CNN and Mask R-CNN, have effectively segmented radiographs for periodontitis detection.^12^^,^^18^^,^^24^, ^25^, ^26^ DenseNet is a CNN architecture that connects all layers feed-forwardly, unlike traditional networks that connect only consecutive layers. This design helps mitigate the vanishing-gradient problem, promotes feature reuse, and improves parameter efficiency, enhancing performance in image recognition tasks.^27^ DenseNet has not been extensively explored to detect oral pathologies in dental radiographs; its potential merits warrant further investigation. This study introduces a novel Two-Stage CNN model integrating Mask R-CNN and DenseNet169 to address this research gap by enhancing detection accuracy and staging precision for periodontitis in panoramic radiographs, thereby offering a robust and innovative approach in dental image analysis.

## 2 Methods

### 2.1 Datasets

A total of 600 panoramic radiograph samples were retrospectively collected from medical records at the Dental Hospital of Universitas Gadjah Mada were digital radiographs obtained using a Vatech Pax-i PCH-500 X-ray machine (Vatech Pax-i Co. Ltd., South Korea) with exposure settings of 90 kV, 10 mA, and 12 s. For training, validation, and testing of the Two-Stage CNN model, the digital panoramic radiographs were exported without patient identity using EzDent-i Vatech software (Gyeonggi-do, South Korea) in BMP format with dimensions of 2868 × 1504 pixels and subsequently converted to.jpg format before being fed into the Two-Stage CNN model.

Panoramic radiographs used in this study had to meet the following inclusion criteria: include images of the lower mandibular border, right and left condyles, and the lower orbital margin; good contrast, detail, and sharpness; absence of distortion, artifacts, and ghost images; and obtained from patients aged 20 years or older with non-crowded permanent teeth. Radiographs depicting pathological conditions in the mandible or those showing deciduous or mixed dentition were excluded.

The sample size was determined by applying Lemeshow's formula for studies with undefined populations: n = [Z_(1-α/2)^2 × P(1-P)]/d^2, with Z = 1.96 (95 % CI), P = 0.85 ^12^ and D = 0.05. This calculation yielded a minimum sample requirement of 196 digital panoramic radiographs. To develop the deep learning model, we distributed 600 radiographs equally across all classes (120 per category, see Table 1). All research samples, consisting of digital panoramic radiographs, were divided into three main groups as the initial dataset: training dataset (70 %), validation dataset (10 %), and testing dataset (20 %). At the end of the experiment, an additional 100 panoramic radiographs were collected and used as external data for final testing for the Two-Stage CNN. The distribution of the dataset in this study is outlined in Table 1.

**Table 1:** Distribution of the dataset.

Group	Training Dataset	Validation Dataset	Testing Dataset	Total Dataset
Normal	84	12	24	120
Stage 1	84	12	24	120
Stage 2	84	12	24	120
Stage 3	84	12	24	120
Stage 4	84	12	24	120
Total dataset	420	60	120	600
Total dataset after augmentation	840	120	240	1200
Percentage	70 %	10 %	20 %	100 %
External dataset for final testing	0	0	100	100

### 2.2 Data annotation

**Fig. 1:**
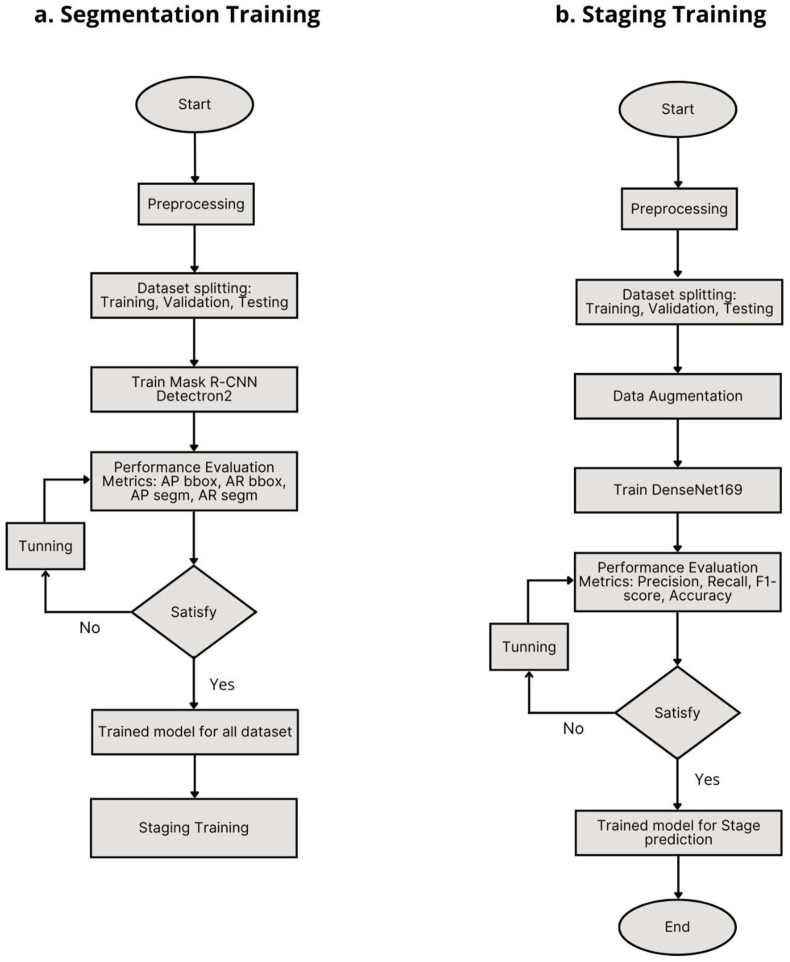
The flowchart of the training process for the Two-Stage CNN used for periodontitis staging prediction consists of two phases: (a) Mask R-CNN segmentation training and (b) DenseNet169 staging training.

The experiment began with radiograph annotation, followed by segmentation training, staging training, validation, and testing. Fig. 1 presents the training workflow of the Two-Stage CNN. This study used four types of radiograph annotations: three polygon-based annotations for teeth, the cementoenamel junction (CEJ), and RBL, as well as periodontitis staging classification. These annotations were applied for both segmentation and staging training. The VIA labeling platform was used for polygon annotation, and annotations were saved in .json format for segmentation. All radiographs were classified according to periodontitis staging, serving as the ground truth for evaluating the deep learning model. Annotations were performed by a dentist (RW) and a dental student (ANF) based on consensus with an oral radiologist (ERA), referring to the 2017 Classification of Periodontal and Peri-Implant Diseases and Conditions.

### 2.3 Hybrid two-stage CNN model

**Fig. 2:**
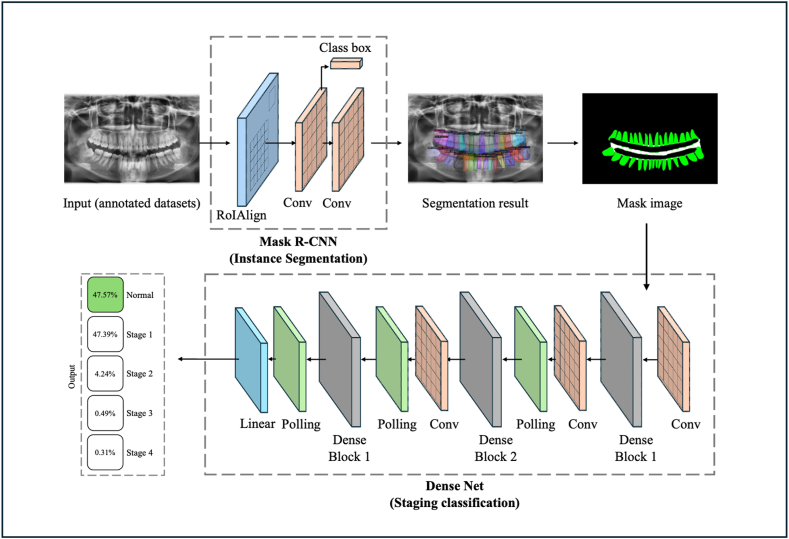
The AI system is based on a Two-Stage CNN, combining Mask R-CNN and DenseNet169, to detect and predict periodontitis in panoramic radiographs.

As its name suggests, the Two-Stage CNN model's training process was conducted in segmentation and staging training (as depicted in Fig. 1, Fig. 2). The segmentation training utilized the Mask R-CNN model, implemented using the Detectron2 framework with a Residual Networks 101 (ResNet101) backbone. This stage focused on training Mask R-CNN to perform three automatic segmentation tasks: segmenting teeth, the CEJ, and RBL. Following this, the staging training employed DenseNet169 to train the Two-Stage CNN model for classifying (staging) periodontitis in panoramic radiographs. The entire training workflow was executed on cloud-based Central Processing Units using Google Collaboratory.

During the segmentation training, parameter tuning, including learning rate (LR) and iteration adjustments, was performed to achieve the best Mask R-CNN model accuracy for identifying masks in each class: teeth mask, CEJ mask, and RBL mask. As outlined in the training workflow in Fig. 1b, the segmentation results (extracted masks for teeth, CEJ, and RBL) were grouped according to their respective stages and subsequently fed into the DenseNet169 model using the PyTorch framework for staging (classification) training.

The mask dataset from the segmentation training was augmented before being used for staging training. The augmentation was performed using horizontal flipping, doubling the dataset size. The original training: validation: testing ratio of 70 %:10 %:20 %, with 420:60:120 samples, was increased after data augmentation to 840:120:240 samples. During the staging training, the DenseNet169 model was trained using various combinations of parameters, including Ratio, ReLU, Dropout, Softmax, and Epoch, which were then tuned to achieve the best-performing prediction model.

### 2.4 Evaluation of diagnostic performance

To evaluate the final performance of the Two-Stage CNN, 100 new radiographs were used for final testing, which had yet to be included in the previous training, validation, or testing phases. These 100 panoramic radiographs comprised images of normal conditions and periodontitis stages 1, 2, 3, and 4, with 20 radiographs for each stage (as outlined in Table 1). The model's performance was assessed using various metrics, including Precision (Positive Predictive Value), Recall (Sensitivity), Specificity, F1-score, and Accuracy. The diagnostic performance of the Two-Stage CNN was quantified using a confusion matrix, following the methodology commonly used in medical diagnostic testing,^28^ as presented in Table 2. The prediction runtime for a single case was assessed in a local testing environment using an AMD Ryzen7 4800H (16 CPUs) @2.9 GHz processor and an NVIDIA GeForce GTX 1660 Ti with Max-Q Design GPU.

**Table 2:** A comparison between the diagnostic test calculations^29^ and the performance evaluation of AI using the confusion matrix.^30^.

Performance Metric	Diagnostic Test	Confusion Matrix
Sensitivity	TP/(TP + FN)	Recall/True Positive Rate
Specificity	TN/(TN + FP)	Specificity = True Negative Rate
PPV (Positive Predictive Value)	TP/(TP + FP)	PPV = Precision
NPV (Negative Predictive Value)	TN/(FN + TN)	TN/(FN + TN)
Accuracy	(TP + TN)/(TP + FP + TN + FN)	(TP + TN)/(TP + FP + TN + FN)
F1-score	Not available	F1-score = 2 × Precision × Recall/(Precision + Recall)

TP = True Positive; FN = False Negative; TN = True Negative; FP = False Positive.

## 3 Result

**Fig. 3:**
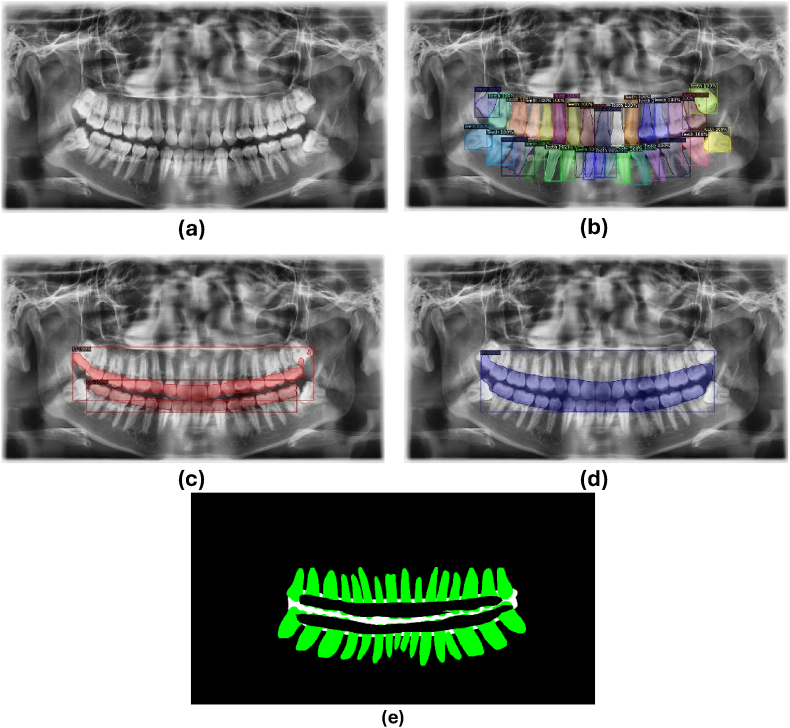
(a) A sample of panoramic radiograph from the dataset, with the results of segmentation training indicating the detection of (b) teeth, (c) the cementoenamel junction (CEJ), and (d) radiographic bone loss (RBL) on the same image. (e) Mask from the segmentation training, which serves as input for the staging training.

Fig. 3 demonstrates the successful segmentation training of Mask R-CNN, which accurately delineated the teeth, CEJ, and RBL regions consistent with the annotations. The segmentation masks (Fig. 3e) were then used for staging training using the DenseNet169 model.

**Fig. 4:**
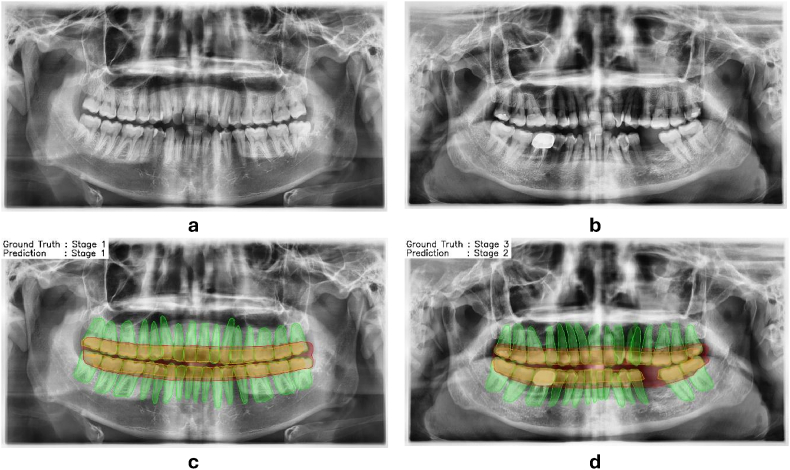
(a) and (b) Panoramic radiographs used for testing the Two-Stage CNN model, with (c) the correct detection results from radiograph (a) and (d) the incorrect detection results from radiograph (b).

Following the validation process, testing was conducted with 120 radiographs (20 % of the sample), as specified in Table 1. Fig. 4 presents sample cases and periodontitis detection using the hybrid Two-Stage CNN model. In Fig. 4c and d, the green areas represent detected teeth, the red indicates RBL, and the yellow shows the detection of the tooth crown up to the cervical or CEJ border. While Fig. 4c shows correct detection according to the ground truth, Fig. 4d demonstrates a misidentification by the model.

Further evaluation of the Two-Stage CNN model was conducted on 100 new radiographs from an external dataset that had yet to be utilized in the training, validation, or testing phases. The initial and final testing (using the external dataset) outcomes of the hybrid Two-Stage CNN are shown in the confusion matrix (Table 3), which is subsequently used to evaluate the model's performance in detecting and staging periodontitis on panoramic radiographs. The summarized results of this performance evaluation of the hybrid Two-Stage CNN are presented in Table 4.

**Table 3:**
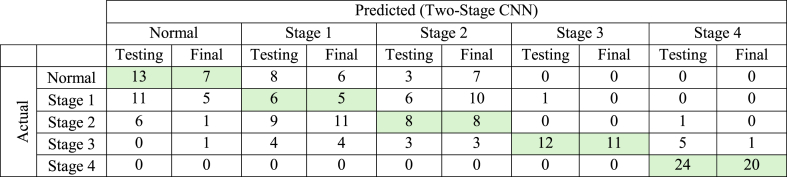
Confusion matrix results from the Two-Stage CNN testing and final testing (using the external dataset) for periodontitis detection on panoramic radiographs.

Green boxes indicate the values of True Positive (TP). The **testing** results were derived from the initial dataset, while the **final** results were obtained through external dataset evaluation.

**Table 4:**
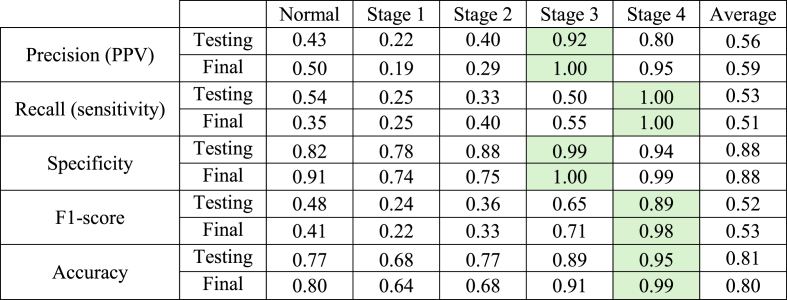
Performance of the Two-Stage CNN for periodontitis detection on panoramic radiographs.

PPV = Positive Predictive Value. Green boxes indicate the best performance in each metric. The **testing** results were derived from the initial dataset, while the **final** results were obtained through external dataset evaluation.

Table 3, Table 4 demonstrate that the Two-Stage CNN consistently performs well in detecting and classifying stage 3 and 4 periodontitis, evidenced by the elevated True Positive (TP) values for stage 3 and 4 periodontitis detection in Table 3. Furthermore, the model exhibits robust performance across all evaluation metrics—including precision, recall (sensitivity), specificity, F1-score, and accuracy—with scores consistently exceeding 0.90 and reaching 1.00 for these stages, as indicated by the green boxes in Table 4. However, the Two-Stage CNN performs poorly in detecting early-stage periodontitis (stages 1 and 2), indicating a need for further optimization. The final testing outcomes (Table 4) reveal the average precision, recall (sensitivity), specificity, F1-score, and accuracy values across all prediction classes of 0.59, 0.51, 0.88, 0.53, and 0.80, respectively. The model achieved its highest performance in periodontitis staging for specificity (0.88) and accuracy (0.80).

The runtime of a deep learning model is a critical factor for clinical application, as it influences the efficiency and feasibility of the model when applied by clinicians. A model with a shorter runtime can improve time efficiency and assist radiologists and clinicians in manual radiodiagnosis processes. In this experiment, the average runtime of the Two-Stage CNN for periodontitis detection on panoramic radiographs was 14.90 s using the CPU and 2.14 s using the GPU.

## 4 Discussion

The application of CNN to detect periodontitis in panoramic radiographs has advanced significantly in recent years. CNN is a DL model originally designed for processing and analyzing visual data.^31^ It consists of interconnected layers of neurons that function to learn and recognize patterns, textures, and features within images.^31^ Due to their diverse structures, CNN models are the predominant deep learning type employed in AI-related oral radiology research, suggesting their superiority in medical image processing.^15^

The two-stage CNN for the detection and staging of periodontitis in this study was conducted sequentially, following the workflow illustrated in Fig. 1. As depicted in Fig. 2, the DL model developed in this experiment integrates two CNN architectures, Mask R-CNN and DenseNet169, which operate stepwise within a single system, hence referred to as the Two-Stage CNN. Mask R-CNN is recognized as an instance segmentation model that effectively segments periodontitis in panoramic radiographs. However, its performance is inferior to other CNN models, such as Multi-Label U-Net, designed for semantic segmentation.^12^ With a trained ResNet-101 backbone, Mask R-CNN has also been successfully implemented for tooth segmentation and numbering in DiagnoCat, a web-based AI software for periodontitis detection.^18^ In the Two-Stage CNN, segmentation is performed using Mask R-CNN with the detectron2 framework and a ResNet101 backbone. This combination of detectron2 and ResNet101 has also been utilized in prior research for detecting periodontally compromised teeth in panoramic radiographs using Faster R-CNN.^26^

The initial stage of the Two-Stage CNN involves segmentation training using Mask R-CNN, producing segmentation results as shown in Fig. 3. The output, a mask (Fig. 3e), is further processed by DenseNet169 to classify periodontitis staging. DenseNet169 acts as a classifier, receiving three segmented masks from Mask R-CNN: teeth, CEJ, and RBL masks, which are used for training. To enhance diagnostic performance, Mask R-CNN is integrated with DenseNet169 for classification. Among deep networks like ResNet50, DenseNet121, and InceptionV3, a prior study reveals that DenseNet121 combined with global average pooling and mRMR feature selection demonstrated superior performance in staging periodontal bone loss.^19^

The Two-Stage CNN effectively performs segmentation and staging, as illustrated in Fig. 4c. However, discrepancies exist between predicted and actual (ground-truth) staging results (Fig. 4d). Table 4 shows that precision was lower for normal conditions and early-stage periodontitis (stages 1 and 2), leading to higher false positive rates. This study's result is consistent with a prior study,^19^ highlighting challenges in distinguishing normal conditions from early-stage periodontitis.

Specificity reflects a model's ability to correctly identify healthy teeth unaffected by periodontal disease, essential for preventing misdiagnosis and unnecessary treatments. In this study, the hybrid Two-Stage CNN achieved a specificity of 0.88 (Table 4), comparable to the 0.88 reported for Fast R-CNN in detecting healthy teeth.^20^ Often paired with sensitivity to calculate AUC, specificity is a key diagnostic performance metric, though sometimes excluded from reports.^15^^,^^17^^,^^32^ A prior study^19^ reported accuracy below 0.80 due to challenges distinguishing periodontitis stages 2 and 3 from stages 1 and 4, compounded by dataset limitations and class imbalance. Consolidating prediction classes into "healthy," "Stage1/2," and "Stage3/4″ improved accuracy to over 90 % using DenseNet121 + GAP + mRMR-based SVM,^19^ though this approach was not applied in the present study. Similarly, the AI-based web application DiagnoCat, designed for detecting teeth and periodontitis, also used modified prediction classes.^18^ Normal conditions and stage 1 were grouped in this previous study^18^ for binary classification as "false." In contrast, stages 2 and beyond were classified as "true," achieving F-scores, accuracy, and Cohen's kappa coefficients exceeding 0.90.

The development of hybrid networks integrating segmentation and classification functions represents a promising solution to enhance the performance of DL for periodontitis classification in radiographs.^12^ Previous study^25^ successfully developed Deetal-Perio, an AI system based on Mask R-CNN, designed for dual segmentation tasks—teeth numbering and alveolar bone segmentation—integrated with XGBoost to predict the severity degree of periodontitis based on alveolar bone loss in panoramic radiographs. This study adopts the training methodology from prior research,^24^ which utilized a hybrid CNN architecture for detection and conventional methods for periodontitis classification in panoramic radiographs. The hybrid CNN model in prior research^24^ employed three types of segmentation on panoramic radiograph datasets: targeting periodontal bone level (PBL), cementoenamel junction level (CEJL), and teeth detection. Although this segmentation approach demands significant annotation efforts, as each radiograph necessitates four annotations (three polygon annotations for training the segmentation of teeth, CEJ, and RBL, along with one annotation for training the periodontitis staging), the resulting annotations are designed to train DL models to emulate clinicians' diagnostic reasoning when assessing periodontal disease in panoramic radiographs. This process aims to enhance the model's performance as a diagnostic tool to support clinical decision-making.

In addition to being adapted in this study, the three-stage segmentation method from prior research^24^ has also been utilized in subsequent studies.^33^^,^^34^ A multi-task learning strategy was implemented by integrating multiple segmentation and classification networks using a U-Net DL model for periodontitis detection.^33^ In the previous study,^33^ primary segmentation tasks—bone area, tooth, and CEJ—were performed, resembling the segmentation tasks in this study. However, while the previous research^33^ employed U-Net for periodontitis detection in intraoral periapical radiographs, this study applies a Two-Stage CNN to the panoramic radiograph dataset. Another integrated DL framework was also proposed by earlier researchers,^34^ combining U-Net and YOLOv5 for periodontitis detection in panoramic radiographs, where U-Net was used to detect CEJ boundaries and bone loss, and YOLOv5 was utilized for teeth detection.

The 2017 Classification of Periodontal and Peri-Implant Diseases and Conditions often results in diagnostic inconsistencies, especially among less experienced clinicians.^35^ Diagnosing periodontal disease is complex, prompting interest in AI to enhance diagnostic accuracy and inter-clinician consistency.^26^ Furthermore, developing DL models to assist in diagnosing periodontitis based on the 2017 World Workshop remains a significant challenge for computer scientists, oral radiologists, and other dental clinicians due to the difficulty in creating accurate DL models for detecting early-stage periodontitis, as reported in this work and previous studies.^18^^,^^19^

In this study, the Two-Stage CNN achieved an average runtime of 14.90 s on a CPU and 2.14 s on a GPU, which is slower than the 0.027 ± 0.002 s per image reported for the PAR-CNN model.^15^ However, the runtime is comparable to or faster than the time clinicians require: 6.042 ± 1.148 s for periodontists and 13.105 ± 3.153 s for general dentists when diagnosing periodontitis on panoramic radiographs.^36^ AI can potentially support radiologists and dentists in faster and more efficient periodontitis detection. Optimizing AI models for radiology requires balancing complexity and runtime. Greater complexity can increase processing time. High-performance GPUs and efficient algorithms are essential for achieving fast and accurate models suitable for clinical use.

While the Two-Stage CNN demonstrates robust performance in advanced periodontitis staging (Stages 3–4) with specificity >0.88, its reduced precision for early-stage detection (Stages 1–2) and suboptimal runtime (2.14s/GPU) highlight critical limitations. Future study should optimize model architecture through multi-task learning strategies (e.g., U-Net/YOLOv5 integration), address class imbalance via staged reclassification, enhance computational efficiency for real-time clinical deployment aligned with the 2017 Classification criteria, and incorporate three-dimensional CBCT imaging datasets to improve diagnostic accuracy and clinical relevance.

## 5 Conclusion

The Two-Stage CNN, a hybrid model integrating Mask R-CNN and DenseNet169, demonstrated robust diagnostic performance for detecting and staging periodontitis in panoramic radiographs, achieving specificity and accuracy of 0.88 and 0.80, respectively. Mask R-CNN functions as an instance segmentation framework to generate teeth, CEJ, and RBL masks, which DenseNet169 subsequently classifies to determine the periodontitis stage. The hybrid two-stage CNN has the potential to be further developed into a computer-aided diagnostic tool for the detection of periodontitis on panoramic radiographs, supporting the management of periodontal disease, which is one of the most prevalent oral diseases after dental caries.

## Patient’s/guardian’s consent

All datasets employed in this investigation comprise anonymized secondary data devoid of personal identifiers. The study received a waiver of consent from the institutional review board due to the absence of direct patient interaction. The study adheres strictly to institutional ethical standards and data protection regulations, with access to datasets limited to the research team.

## Ethical considerations

The ethical approval for the investigation was bestowed by the Ethics Committee associated with the Faculty of Dentistry and Prof. Soedomo Dental Hospital at Universitas Gadjah Mada (Reference No. 72/UN1/KEP/FKG-RSGM/EC/2024).

## Funding disclosure

This study received financial support from the Faculty of Dentistry at Universitas Gadjah Mada, Indonesia (Grant Number: 3877/UN1/KG/Set.KG1/LT/2024).

## Declaration of competing interest

The authors declare no conflict of interest concerning this publication.

## Data Availability

The dataset comprising panoramic radiographs utilized in this investigation is not available for public access. Nevertheless, it may be granted upon a reasonable request.
